# Production and Evaluation of Synthetic Lightweight Aggregates Based on Mixture of Fluidized Bed Fly Ash and Post-Mining Residues

**DOI:** 10.3390/ma15020660

**Published:** 2022-01-16

**Authors:** Grzegorz Skotniczny, Mateusz Kozioł, Jerzy Korol, Paweł Poneta

**Affiliations:** 1 Limestone Mine “Czatkowice” sp. z o.o., ul. Czatkowice Dolne 78, 32-065 Krzeszowice, Poland; skotniczny@czatkowice.com.pl; 2 Faculty of Materials Engineering, Silesian University of Technology, ul. Krasińskiego 8, 40-019 Katowice, Poland; 3 Central Mining Institute, Plac Gwarków 1, 40-166 Katowice, Poland; jkorol@gig.eu; 4 Tauron Polska Energia S.A., ul. ks. Piotra Ściegiennego 3, 40-114 Katowice, Poland; pawel.poneta@tauron.pl

**Keywords:** lightweight aggregate, mining by-products, fluidized bed ash, physical properties, mechanical properties

## Abstract

This paper presents an attempt to obtain technically valuable lightweight aggregate produced from a mixture of fluidized bed fly ash and post-mining residues. The motivation to take up this study is a problem with the reasonable utilization of huge amounts of ashes produced by power plants in Poland. The ashes still produced and those stored in heaps amount to a tonnage of millions, and new ways to utilize them are desired. A real lack of mineral aggregates (non-renewable resources) demands the search for alternative materials. Using the industrial ashes as aggregates is a possible solution to the two above-mentioned problems. The aim of the study was to produce the lightweight aggregate components and to assess them in terms of their physical and mechanical properties. The components were prepared by mixing, granulation, and sintering at the temperature of over 1170 °C. Evaluation of physical parameters was based on parameters such as bulk density and water absorption. The study of mechanical properties was carried out on the basis of aggregates’ resistance to crushing. The obtained results revealed that using a mixture of the combustion and post-mining residues in the production of a lightweight aggregate is beneficial and results in the formation of a porous and durable structure. The measured resistance to the crushing of the produced aggregates varied from 5.9 MPa to 7.5 MPa. They also showed a high freeze-thaw resistance and good resistance to aggressive environments (bases, acids, salt). The registered properties indicate that the aggregates meet the basic requirements for materials used in construction and road-building. This study has a scientific and didactic value in that it describes the step-by-step process of planning and implementing the production of synthetic mineral aggregates.

## 1. Introduction

Lightweight aggregates is a definition for porous mineral aggregates with a density less than 2000 kg/m^3^ and a bulk density up to 1200 kg/m^3^ (according to PN-EN 13055:2016-07 [1]). Nowadays, lightweight aggregates can be classified into several groups depending on the type of raw materials used for production: a group of aggregates of natural origin from porous rocks, a group of aggregates of natural origin from thermally processed rocks, a group of artificial aggregates from thermally processed industrial wastes or by-products [2,3,4,5]. The lightweight aggregates produced from industrial by-products or waste materials account for an increasing contribution to building technologies, which fulfils the principles of the circular economy [6,7,8,9,10]. The properties of lightweight aggregates result from their porous structure, due to technological processes. It is mainly thermal processes that result in the formation of air holes enclosed in the structure of individual aggregate grains [11,12,13]. Depending on the raw material used for production, a product with varied properties and different applicability is formed [14,15,16,17]. Fluidized bed fly ash is a mass waste of the electric energy production process and is a serious problem due to the amount generated and moderate toxicity. Nowadays, a significant part of the tonnage is managed in various ways, e.g., to produce bricks [18] or for replacing Portland cement in concrete mixtures [19,20,21,22]. Post-mining residues are also a problem due to the enormous tonnage produced. For some time now, instead of simple storage, valuable raw materials have been recovered from them [23] or they have been used for various industrial purposes [24], including as additives to aggregates [25]. The sustainable management of both fluidized bed fly ash and post-mining residues is an important element of the circular materials economy which has become increasingly important and remains a subject of intense research across various disciplines [26,27,28].

The subject of this study is the assessment of the possibility to produce lightweight aggregates from post-mining residue products and fluidized bed fly ash (power plant post-combustion residue product) occurring in Tauron Polska Energia S. A. (Poland), a big company which owns mining plants and thermal processing power plants as well. The coal mining and power industry based on the combustion of coal will generate significant amounts of mining by-products and combustion by-products in the next 20 years. After this period, most of the fuel combustion plants will be shut down as planned. In addition, great amounts of these types of wastes are stored in heaps, which is dangerous for groundwater, and thus (in some cases) may occasion the necessity of recycling this resource. The solution presented in this paper allows for the economic use of waste, reducing its negative impact on the environment due to its storage. The solution meets the principle of the circular economy. Additionally, it has potential to reduce the depletion of non-renewable natural aggregate resources.

**Figure 1:**
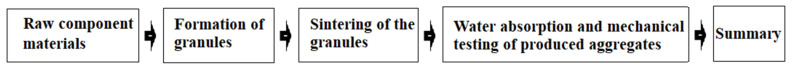
Scheme of the study—subsequent stages of the obtaining and testing aggregates.

This study describes the production process and evaluation of aggregates composed from three large-volume mineral wastes: fluidized bed power plant fly ash (as the main evaluated component), coal sludge, clay from washing raw limestone bulk. The compositions were evaluated in two main scopes: (1) from a technological point of view, focusing on ease and efficiency of manufacture, and (2) for applicability estimation. In particular, the effect of the fluidized fly ash content in the compositions was assessed. A wide range of research instruments was used in the evaluations, including thermal and microscopic analyses, while all conclusions are both practical and scientific. The presented process for producing aggregates is the subject of a patent application to the Patent Office of the Republic of Poland. This study is divided into sections concerning the subsequent stages of obtaining and testing aggregates, as presented in Figure 1.

## 2. Raw Component Materials

The raw component materials for producing aggregates were fluidized bed fly ash, clay (waste remaining after washing raw limestone bulk), and coal sludge. The components are described in Table 1.

All samples of components for testing were taken directly from production bulk buffers (silos or stockpiles) during a stable technological process. Thus, representative samples of raw materials were obtained. In total, 500 kg of PF, 1000 kg of GC, and 1500 kg of SW have been captured for testing. Chemical analysis of the raw materials was performed by X-ray fluorescence (XRF) using an Axios mAX spectrometer (manufacturer Malvern Panalytical, Malvern, UK). In PF, quartz, dehydroxylated silt loam minerals, and anhydrite were found. In GC, calcite, serpentinite clay minerals, and quartz were found. In coal slime, apart from coal, quartz and silica minerals were found. Chemical compositions of the studied raw materials are listed in Table 2. It should be emphasized that in addition to the compositions indicated, the materials contained carbon. The used measurement method focused on the precise determination of the remaining compounds.

**Figure 2:**
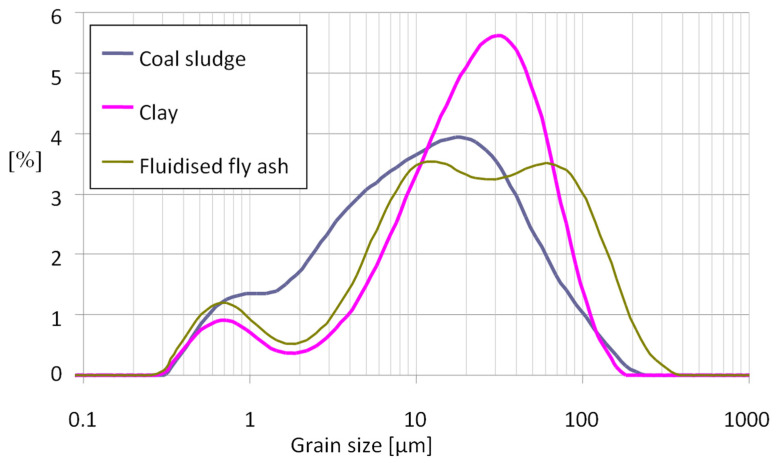
Mass fraction vs. grain size distribution relation determined for the raw components.

The particle size distribution of the raw components was determined with use of Mastersizer 2000 laser scanner made by Malvern Panalytical (Malvern, UK). The test was conducted in a wet medium, water. Test findings in the form of a mass fraction vs. grain size relationship are shown in Figure 2.

The analysis of grain composition shows that it is in the range from 0.4 to 400 µm, but the largest proportion by far is in the range from 5 to 100 µm.

In order to determine the thermal properties of the raw components, DTA/TG analysis and chemical analysis of the gases emitted during thermal processing were carried out. The practical purpose of the analyses is to address two salient issues.

(1) The extent of thermal transformation and the temperature at which this transformation proceeds for each of the tested components. This is necessary for setting the parameters of sintering.
(2) The impact of the production process on the external environment.

**Figure 3:**
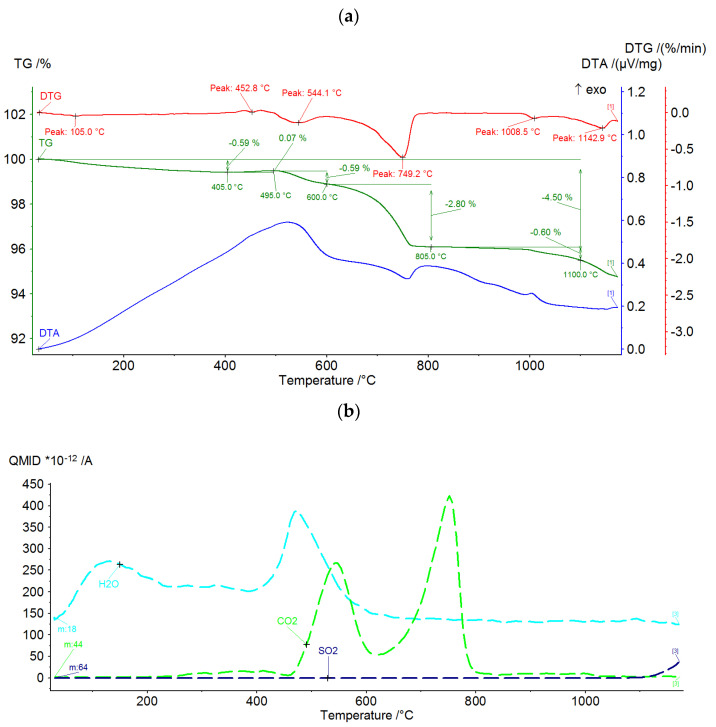
DTA/DTG curves (**a**) and gas analysis (**b**) during thermal treatment of fluidized bed fly ash PF.

**Figure 4:**
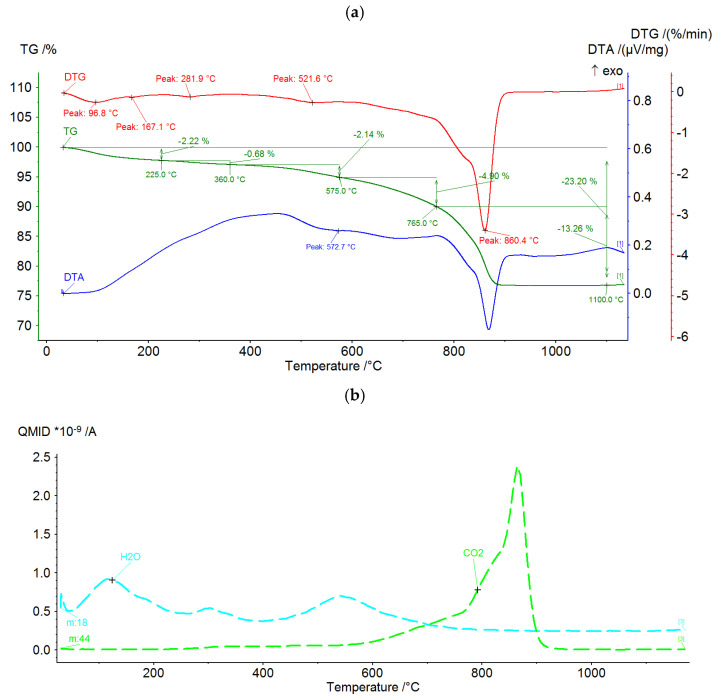
DTA/DTG curves (**a**) and gas analysis (**b**) during thermal treatment of clay GC.

**Figure 5:**
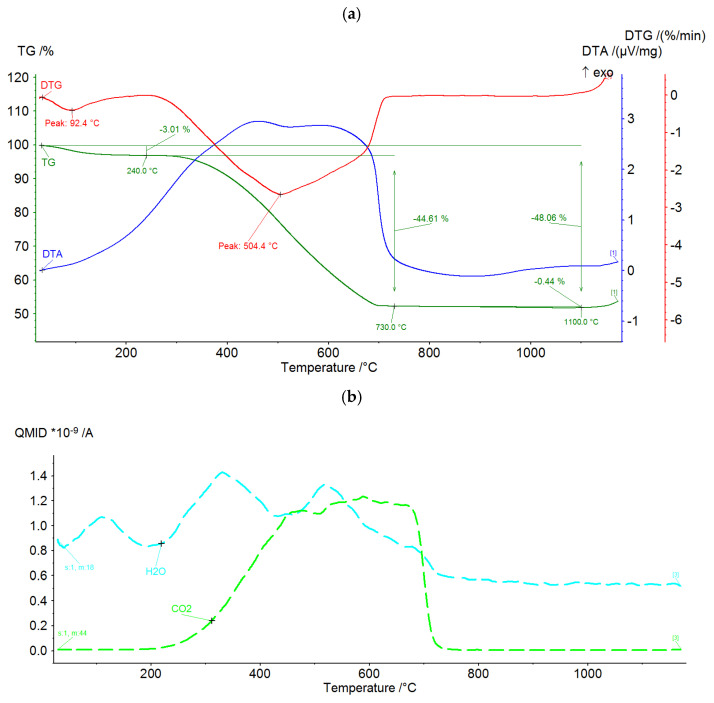
DTA/DTG curves (**a**) and gas analysis (**b**) during thermal treatment of coal sludge MW.

A Netzsch STA 449F3 Jupiter (Selb, Germany) was used to perform the DTA/DTG tests. The test was conducted in a synthetic air atmosphere at a flow rate of 40 mL/min. The heating rate was 15 °C/min. Feedstocks dried at 105 °C and ground to a grain size below 0.06 mm ranged from 85 to 95 mg. Thermal and chemical test results are shown in Figure 3, Figure 4 and Figure 5. Most of the thermal transformations were found to take place in all three components tested below 900 °C. In the case of the ash (PF) above 1000 °C, a progressive weight loss begins, overlapping approximately with the release of SO_2_. This probably involves oxidation of previously dehydrated sulphates. In the case of clay (GC), CO_2_ is emitted intensively before 800 °C. This is due to the decomposition of calcium carbonate. Sludge (MW) shows the intense emission of CO_2_ in the range of 300–700 °C, well overlapping with the increased level of DTA. This is undoubtedly related to the intensive oxidation of the carbon contained in the structure. It can be presumed that all three components will show significant physicochemical activity at temperatures above 1000 °C.

High-temperature microscopy represents a group of thermal methods in which the measurement is based on recording changes in the shape of the sample during its heating. Characteristic temperatures are determined during measurement. The most significant are:

− temperature of (first sinter-related shrinkage),
− maximum sintering temperature (maximum sintering shrinkage with no deformation),
− softening temperature (first deformation of the sample—the corners round),
− temperature of maximum expansion,
− melting point temperature (sample takes hemispheric shape).

The test was performed on a sample made from a mixture of the three components, of the following composition: PF—20%, MW—40%, GC—40%. The mixture was prepared by mixing them and pressing into 3 × 3 mm cylinder-shape samples with a pressure of 90 MPa. Fractions of the components of grain size <0.063 mm were used. The contents of individual ingredients were determined on the basis of previous practical experiments in aggregate production.

**Figure 6:**
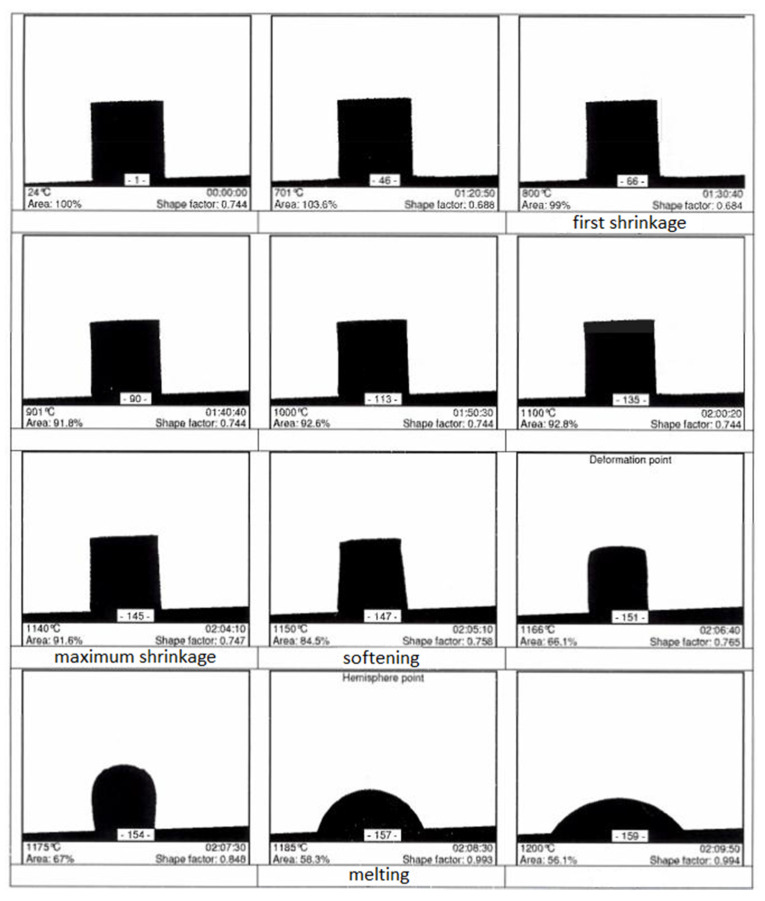
High temperature microscope test results for composition of PF (20%), MW (40%) and GC (40%).

A high-temperature microscope made by Hesse Instruments (Osterode am Harz, Germany) was used. Measurements were carried out in an air atmosphere with a constant heating rate of 10 °C/min to a temperature of 1400 °C. Measurement results, in the form of photographs of the sample outlines at different stages of the test, are shown in Figure 6.

Obtained test results indicate that, at the assumed proportions of particular components, effective sintering, considering heat losses during the real process and heat transfer limitations due to the thermal insulation of components, will require a temperature of 1200 °C inside the sintering chamber.

## 3. Formation of Granules

Taking into account previous experience with the preparation of aggregates composed from various industrial wastes, three combinations of the studied ingredients were selected for production of the pre-sintered aggregate grains, i.e., granules (as bulk called “granulates”). Early experiments pointed to the advisability of adding maximum 20% by weight of PF. The selected compositions are shown in Table 3.

**Figure 7:**
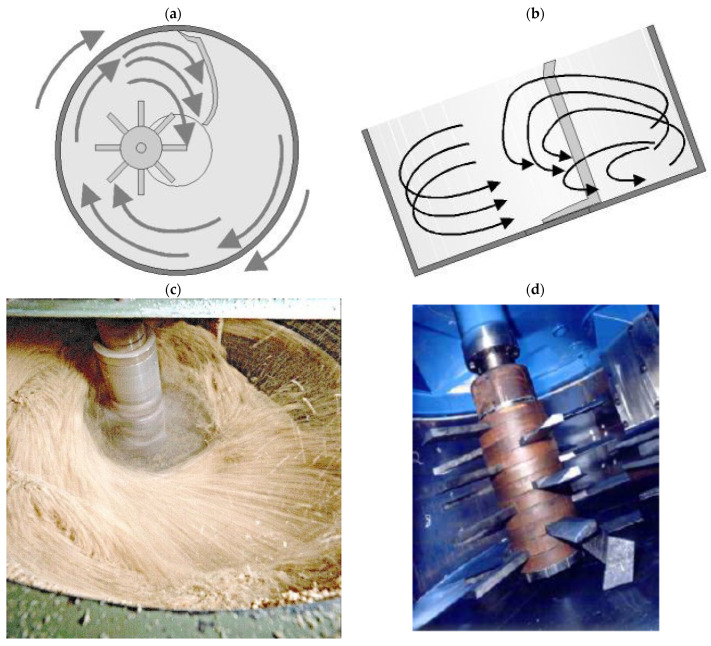
Intensive counter-rotating mixer used for homogenizing the mixture of raw components and for joining them into granules: (**a**) scheme with marked directions of the mixed mass movement—top view, (**b**) scheme with marked directions of the mixed mass movement—side view in section, (**c**) actual picture of the mixer during operation, (**d**) star belt type impeller.

Forming granules requires two effects to occur in succession during mixing: homogenization of the components and their joining, i.e., granulation. The mixing process is carried out until the appropriate grain size is obtained, as initially assumed for the given raw material composition. To achieve the combined effect of homogenization and granulation, the components are mixed intensively in a mechanical mixer. An industrial device with a bowl capacity of 400 dm^3^ (produced by IdeaPro, Nowa Sól, Poland) and a specially designed impeller (star belt type) and counter-rotating scraper, moving in the direction of the mixer rotation, was used in the study. The linear velocity of the blade tips at a rotational speed of 600 rpm was approximately 3 m/s. Due to the relatively high stirring speed of up to 1000 rpm and the high intensity of mixing, the mixer is called an “intensive mixer”. Schematic images of the equipment are shown in Figure 7.

Mixing, homogenizing, and granulating in a counter-rotating intensive mixer is a modern, high-performance way of pretreating fine-grained raw materials. Originally, it was important to adopt a mixing and granulation method that is as simple, reliable, accurate, and industrially applicable at high capacities as possible. Granulation during intensive mixing takes place as a result of the local dynamic pile-up and compression of components’ grains, which are bound to one another mainly by mechanical sticking and, to a minor extent, by local adhesive bonding. Using a counter-rotating intensive mixer for granulation provides a very wide range of possibilities for controlling the granulation process and thus the properties of the final product. With parameters such as the rotor speed, process time, and moisture content of the granulated mass, it is possible to control the final granulation.

**Figure 8:**
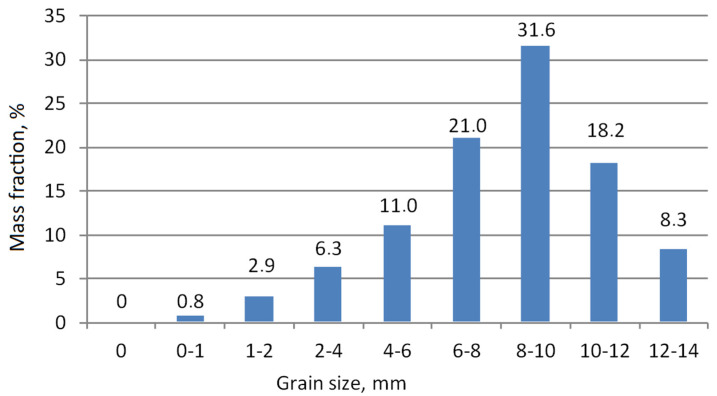
The content of individual grain fractions in the produced granulate.

**Figure 9:**
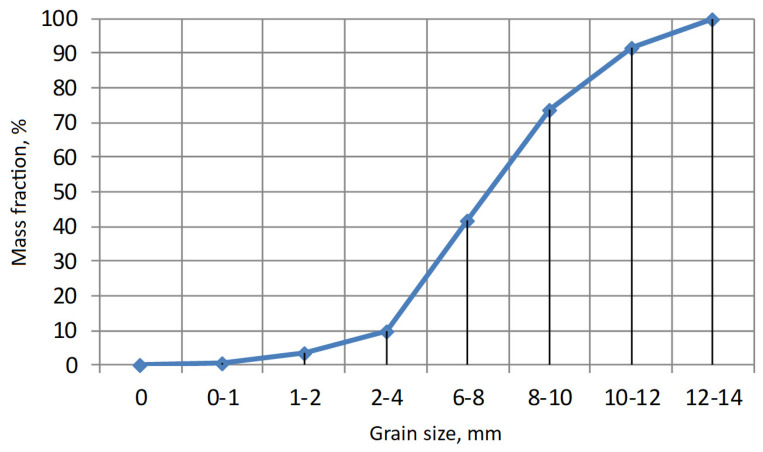
Cumulative grain size distribution curve of the produced granulate.

As a result of the granulation process, granules with different grain size were obtained for different compositions. The target was to obtain granules with a main diameter of about 10 mm. The time of the mixing process required to obtain such a grain size was from 3 to 5 min. Figure 8 shows the grain composition of the granulate obtained, and Figure 9 shows the cumulative distribution curve.

The obtained grain fraction composition indicates an approximately 50% share of fractions within the range of 8–12 mm, i.e., assumed to be approximately 10 mm. Only a very small (less than 1%) share of fractions smaller than 1 mm was observed. This is an advantageous effect, taking into account the further processing of granulate (sintering) and final destination of the produced aggregate in construction and road engineering.

## 4. Sintering of the Granules

Following granulation, the formed granules were sintered. This process was performed at the Łukasiewicz Research Network, Institute of Ceramics and Building Materials, Glass and Building Materials Division in Kraków, Poland. A lab-scale tubular furnace, 0.4 m in diameter, 7 m long, and heated with fuel oil (ICaBM’s own design and production) was used. The process temperature was 1200 °C, and process time 0.3 h. Raw granulates were sintered into form of aggregates with expected properties equivalent to lightweight aggregates. The bulk density of the aggregates obtained, for the three component mixtures used, is shown in Table 4.

To identify the phase composition of the obtained aggregates, roentgenographic tests were performed using the Debye–Scherrer powder method. An X-ray diffractometer (Philips X’PERT PW 3020, Philips, Amsterdam, the Netherlands) was used in the study. The results are presented in the form of a written description (the diagrams have not been registered). The thermal decomposition of coal sludge and clay together with fly ash components resulted in the formation of silicate and aluminosilicate compounds. Fly ash (PF) contains compounds referred to as active silica (SiO_2_act) and active alumina (Al_2_O_3_act). Silicate and aluminosilicate compounds, including calcium and magnesium silicates (Ca,Mg)(Si,Al)_4_O_8_ corresponding to diopside, were identified in all sintered granules tested. The clay (GC) contribution resulted in the formation of wollastonite (CaSiO_3_) and the coal sludge (MW) resulted in the formation of iron-rich calcium silicates of the hedenbergite type (Ca,Fe (Si_2_O_6_)_2_). Apart from the aluminosilicate compounds, potassium aluminosilicate (K(Si_3_Al)O_8_) corresponding to sanidine and mullite 3Al_2_O_3_ ∙ 2SiO_2_ are present. Such observations are reproducible for all three aggregate types (G1, G2, G3).

**Figure 10:**
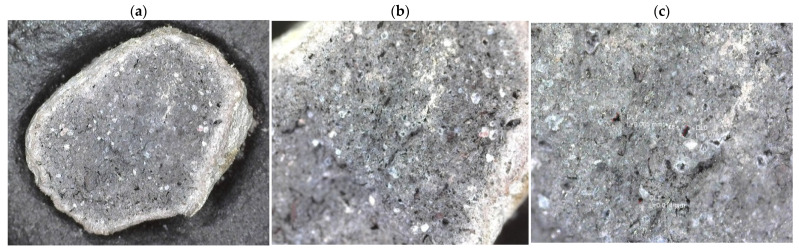
Fracture surface of an aggregate grain after sintering, magnification: (**a**) 30×; (**b**) 70×; (**c**) 175×.

**Figure 11:**
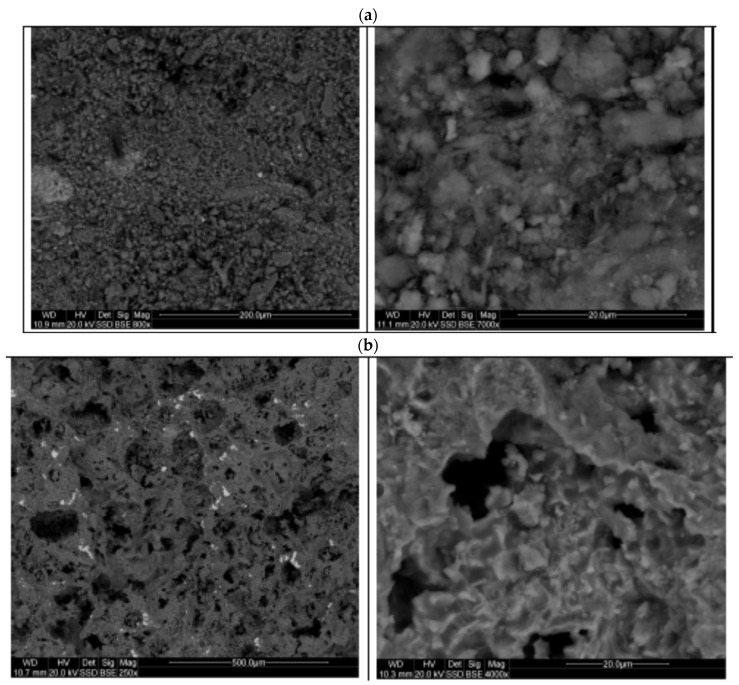
Microphotographs of granulate grain’s fracture surface before sintering (**a**) and after sintering (**b**).

The effectiveness of the sintering process was assessed by evaluating the microstructure of the obtained aggregates. A photograph of the fracture of the aggregate grain after sintering is shown in Figure 10 (Microscope: Olympus BX43). Exemplary micrographs of granules before and after sintering, taken with a scanning electron microscope (FEI Quanta 200FEG, FEI, Hillsboro, OR, USA), are shown in Figure 11.

The photographs shown in Figure 9 indicate a compact grain structure, with a clear difference in colour between the inner and outer surround surfaces. The porosity is predominantly fine, with few large pores and no significant large cracks. Scanning microscope images (Figure 11) revealed the open porous structure of the granules after sintering. Images confirm the effectiveness of the sintering process under the applied conditions. The entire structure is compact, and no fine-grained elements separated from it are observed.

## 5. Water Absorption and Mechanical Testing of Produced Aggregates

Due to the intended application of the tested aggregates in the construction industry, the crushing resistance was considered as the most important functional parameter for evaluation. Testing resistance to crushing was carried out according to the PN-EN 13055-1 [29] standard.

Three samples were prepared from each aggregate for crushing resistance tests. According to PN-EN 13055-1, the crushing resistance test can be performed for aggregates with a grain size between 4 mm and 22 mm and a bulk density greater than 150 kg/m^3^.

**Figure 12:**
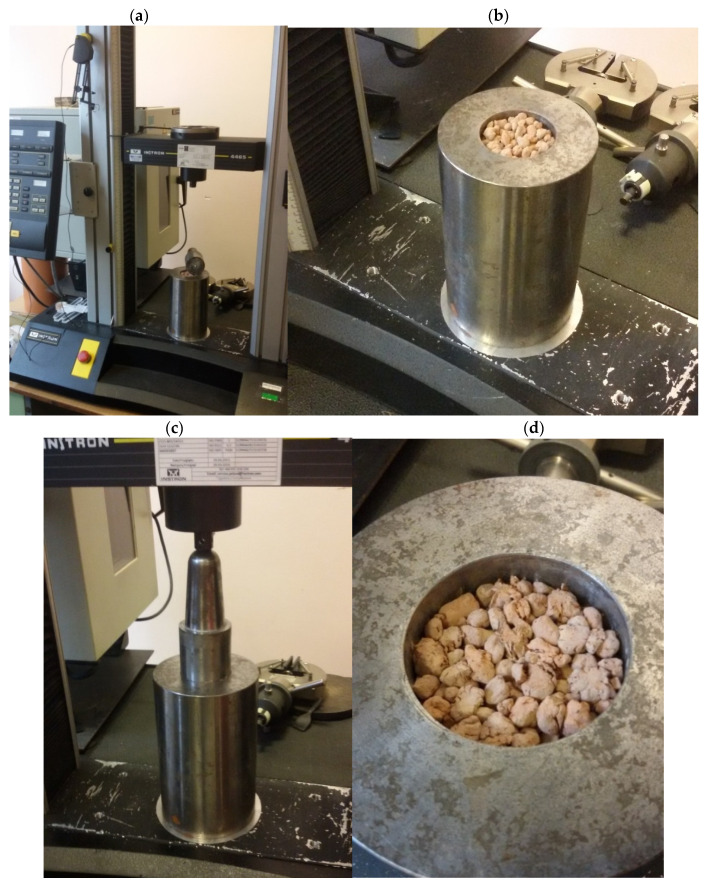
Testing stand for crushing of aggregates: (**a**) testing machine INSTRON 4469, (**b**) aggregate sample in steel cylinder before testing, (**c**) sample during loading, (**d**) sample after crushing test.

Therefore, the fraction below 4 mm and the fraction above 10 mm were removed from the samples prepared for the tests of separate aggregates. Aggregate crushing resistance testing consisted in the uniaxial compression of granules portion backfilled into a steel cylinder, using an INSTRON 4469 testing machine (Instron, Norwood, MA, USA). The compression was carried out up to a displacement of 12 mm (to be checked), while load–displacement correlations were recorded. The test stand and an example of a crushing test specimen are shown in Figure 12.

Values of crushing resistance *C_a_* of individual aggregates were calculated from the equation:

$$C_{a}=\frac{L+F}{A}\;N/mm^{2}$$

where:

*C_a_*—crushing resistance, MPa (N/mm^2^),
*L*—force exerted by the piston in the initial position, N,
*F*—force needed to plunge the piston to 12 mm, N,
*A*—piston area, mm^2^.

On the basis of the conducted tests, the crushing resistance of particular tested synthetic aggregates (G1, G2, G3), and additionally one raw granulate (G2—mass fraction 20% of PF, 40% of MW and 40% of GC), was determined in order to determine the efficiency of the sintering process. For the purpose of describing the results, the test samples were designated as follows:

G0—raw granules,
G1—sintered granulate 1,
G2—sintered granulate 2,
G3—sintered granulate 3.

**Figure 13:**
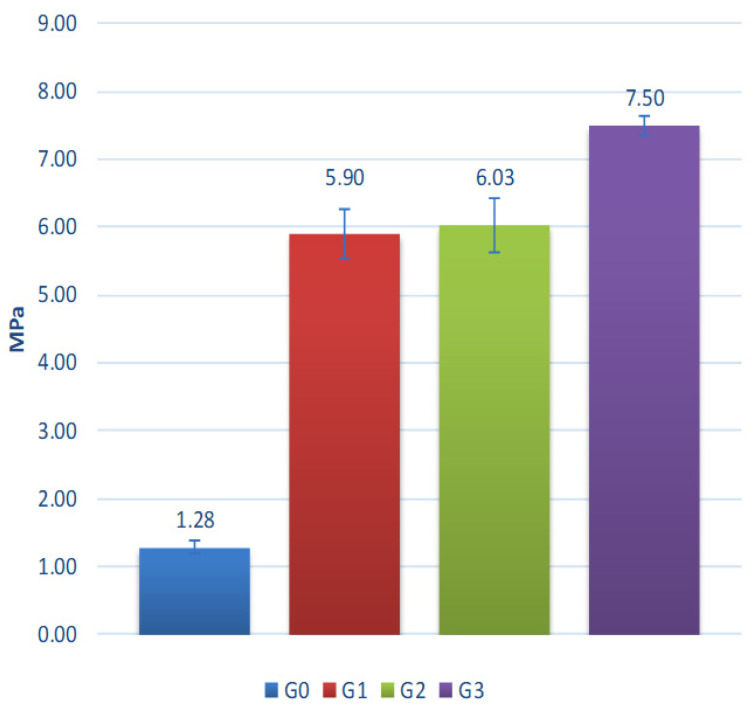
Crushing resistance of the tested aggregates.

Results of aggregate testing without additional exposition of degradation factors are presented in Figure 13.

The crushing resistance of aggregates ranges from 5.9 MPa to 7.5 MPa. It increases with clay proportion and decreases with the proportion of coal sludge in the composition of tested aggregates. The aggregates made of clay or containing predominantly clay in their composition are characterized by the highest strength, due to their most compact structure and minerals formed as a result of sintering. One of the key parameters determining the strength of the sintered materials was the generation and emission of gaseous compounds during the sintering of granules and, as a result, increasing porosity, as proven by the lower bulk density value of sinters obtained in granules with the highest coal sludge content. Coal sludge can be successfully used as an ingredient in the production of lightweight aggregates. However, it is crucial to define the expected correlation of porosity and strength. In the case studied, the reduction in strength is significant with a 20% increase in sludge content resulting in over 23% deterioration of aggregate crushing resistance. Fluidized fly ash has no negative affect on the mixing and granulation process, sintering process, or quality of the aggregate produced. The obtained properties of studied aggregates are comparable with aggregates based on lightweight fly ash described in previous studies [30,31,32].

Summarizing the results of the crushing tests, it should be stated that the resistance of the achieved aggregates is significantly higher or comparable to most aggregates available on the market. For example, the crushing resistance of commercial aggregates tested under similar conditions to those used in the study is:

▪ Liapor—0.7–10 MPa [33],
▪ Arlita—0.98 MPa [34],
▪ Lytag—0.43 MPa [35],
▪ LECA i Ardelite—0.09 MPa [19],
▪ Geokeramzyt Matrix—0.8 MPa [36],
▪ LECA Gniew—0.7–4.0 MPa [37].

For construction and road aggregates, resistance to basic environmental conditions is a very important feature. In order to determine the influence of selected environmental parameters on the properties of aggregates, aggregates were exposed to the following factors:

1. Water absorption and freezing. First, samples of all three aggregates (G1, G2, G3) were seasoned in water for seven days (168 h). Water absorption of these samples was determined. After the water absorption measurements were completed, 10 four-hour freezing cycles at −10 °C followed by thawing at room temperature were carried out for each sample.
2. Soaking in 10% aqueous NaCl salt solution for seven days.
3. Soaking in a 10% aqueous H_2_SO_4_ sulphuric acid solution for seven days.
4. Soaking in a 10% aqueous alkaline NaOH solution for seven days.

**Figure 14:**
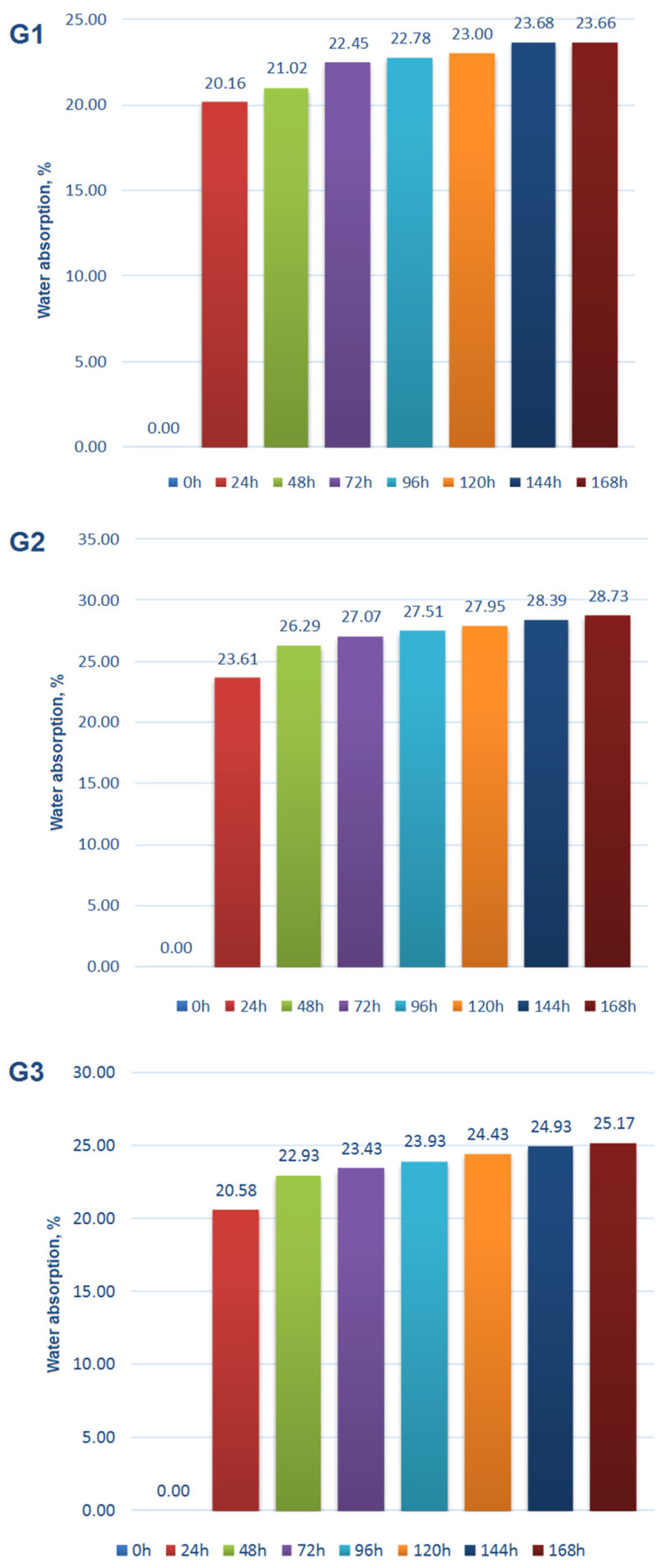
Water absorption of the tested aggregates G1, G2, G3.

**Figure 15:**
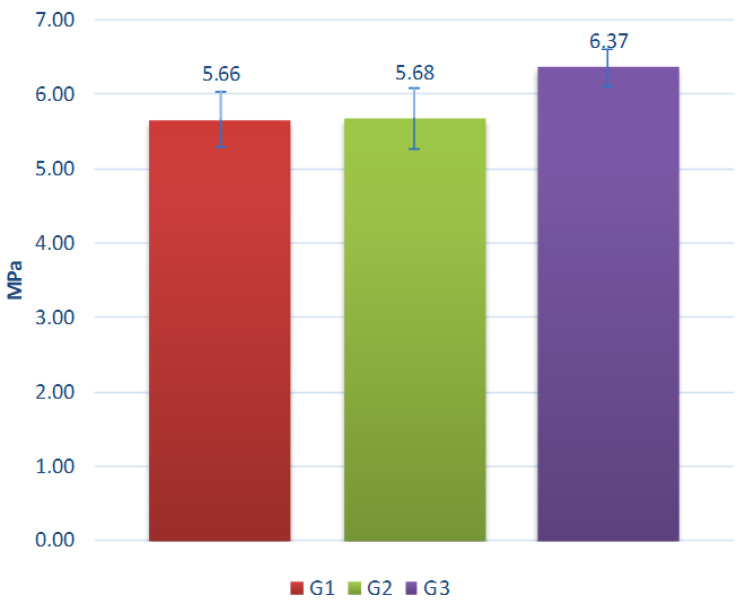
Crushing resistance of the aggregates after cyclic freezing treatment.

**Figure 16:**
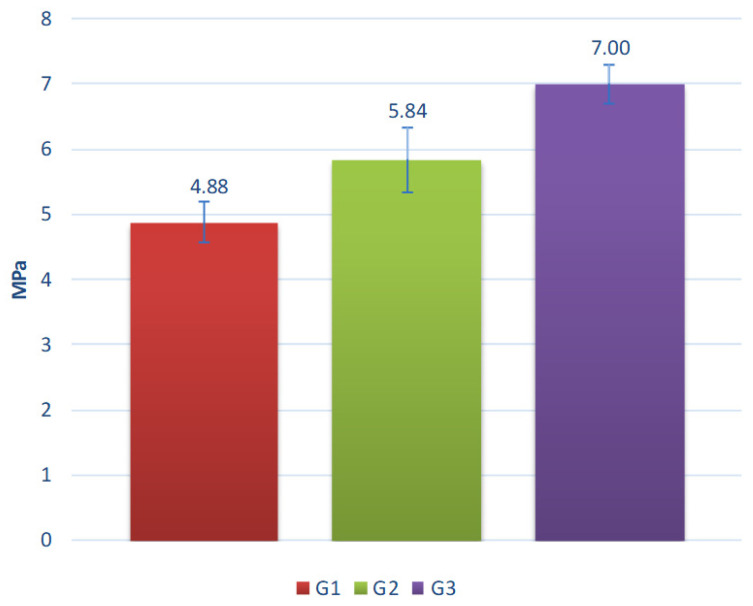
Crushing resistance of the aggregates after on 10% NaCl water solution.

**Figure 17:**
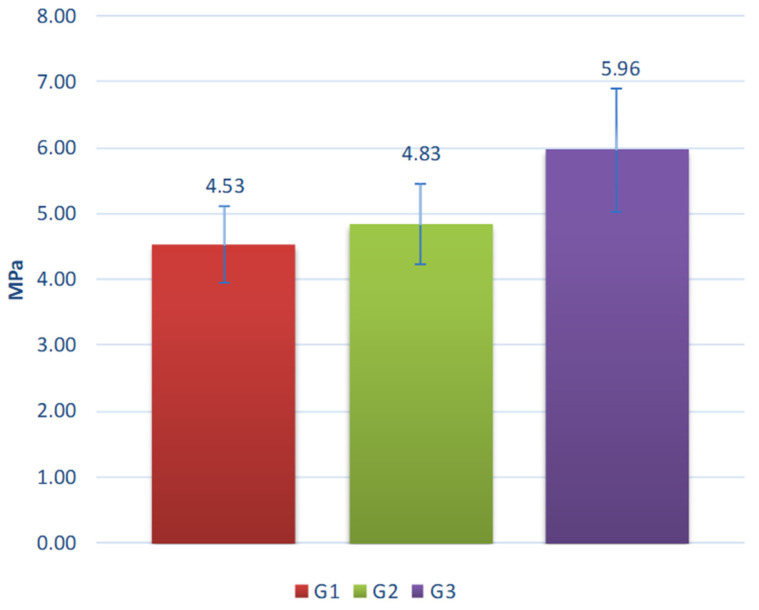
Crushing resistance of the aggregates after on 10% NaOH water solution.

**Figure 18:**
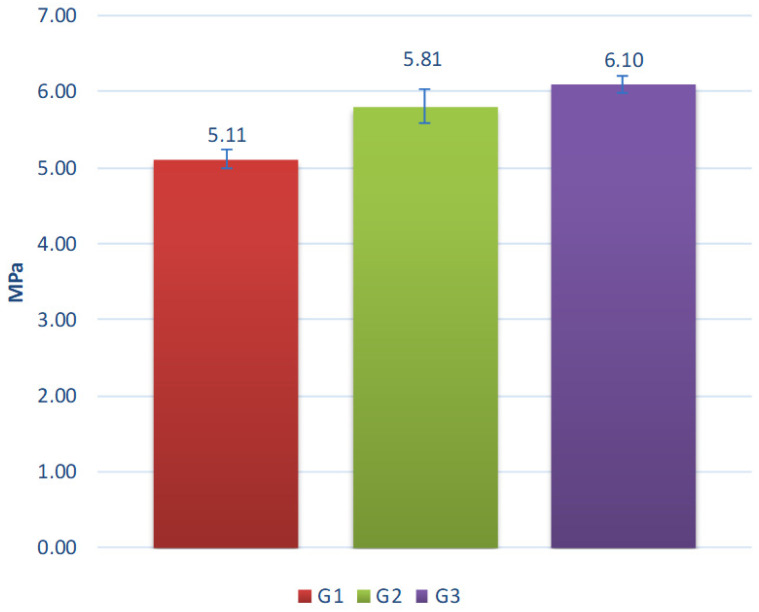
Crushing resistance of the aggregates after on 10% H_2_SO_4_ water solution.

The results of the tests are shown in Figure 14, Figure 15, Figure 16, Figure 17 and Figure 18.

Comparing the results of water absorption tests, it can be stated that the highest water absorption was observed for aggregate G2. It contained an average amount (40% and 40% respectively) of the coal sludge and clay. This indicates that a dominant porosity type in the aggregates is a closed porosity, as supported by the lack of a correlation between the bulk density (Table 4) and the water absorption (Figure 14). The results of crushing resistance of aggregates after cyclic freezing indicate that the tested materials have a relatively good resistance to freezing at high moisture content, with the reduction of parameters being 4%, 6%, and 15% for G1, G2, and G3 respectively. The clay, which is included in 50% in the G3 composition, probably forms some brittle phase within the aggregate during sintering process and the phase is conducive to the occurrence of microcracks during cyclic freezing. The microcracks deteriorate the strength of the material and the increasing volume of clay increases the deterioration. Such a brittle phase may be mullite (one of the main phases occurring in porcelain), produced during sintering compositions containing clay [38].

Evaluation results concerning the crushing resistance of the samples after seasoning in various chemical conditions indicate that seasoning in the bath of alkaline 10% aqueous NaOH solution had the greatest negative influence on the strength. For all aggregates, the decrease in resistance to crushing was approximately 15–23%. For the acid solution, the decreases ranged from 3% to 19% and for the salt solution from 3% to 17%. Composition G1 appears to be particularly sensitive to NaOH solution and to NaCl. This composition contains the highest amount (50%) of the coal slag among the tested materials. Presumably, the chloride ions contained in it (see Table 2) remained within the material after sintering and reacted intensively with Na^+^ ions contained in the NaCl and NaOH solutions. In turn, the G3 composition is especially susceptible to degradation in the H_2_SO_4_ acid. This can be explained by the high calcium content (see Table 2) in clay, which is the main component of the composition (50%). Calcium (even when chemically bound) is highly reactive with strong acids such as H_2_SO_4_.

## 6. Summary

Analyses performed within this study revealed that by-products of mining and combustion, such as coal sludge or clay from washing limestone aggregates, together with fluidized bed fly ash, can be used as resource materials for lightweight aggregates. The resulting synthetic aggregates meet the basic requirements for materials used in the construction industry. Depending on the proportion of raw component materials, the aggregates are characterized by high resistance to crushing, from 5.9 MPa to 7.5 MPa. The high freeze-thaw resistance of the aggregates and their good resistance to aggressive environments prove the potential for their industrial utilisation. Among other things, these aggregates can be used to manufacture lightweight concretes for building and road construction due to their relatively low bulk density.

This study also has scientific and didactic value in that it describes the step-by-step process of planning and implementing the production of synthetic mineral aggregates.

## Figures and Tables

**Table 1:** Raw material components used within the study.

Raw Material	Designation	Short Description	Amount Produced by Tauron Polska Energia
Fluidized bed fly ash	PF	A dry, fine, brown-gray powder	Over 20 thousand tons/year *
Clay (waste remaining after washing raw limestone bulk)	GC	Wet, fine-grained material with a clay-like character and a yellow-sand color	Over 20 thousand tons/year *
Coal sludge	SW	Wet, fine-grained material with a carbon-like character and color	Over 20 thousand tons/year *

* precise data is a trade secret of the company.

**Table 2:** Summary of chemical compositions of raw components used in the study.

Ingredient	Content [% *w/w*]		
	Fluidized Bed Fly AshPF	Clay GC	Coal Sludge MW
SiO_2_	44.23	42.77	53.37
Al_2_O_3_	25.48	14.76	28.03
TiO_2_	1.08	0.79	1.38
Fe_2_O_3_	4.70	5.63	4.96
CaO	13.39	30.91	0.89
MgO	1.88	1.67	1.43
K_2_O	1.89	2.03	3.42
Na_2_O	1.36	-	1.63
SO_3_	5.20	0.13	2.57
Cl^-^	0.21	0.02	1.75
Other	0.58	1.29	0.57

**Table 3:** Compositions of mixtures for the production of the pre-sintered aggregate granulates.

% *w*/*w*	Granulate G1	Granulate G2	Granulate G3
Clay (GC)	30	40	50
Sludge (MW)	50	40	30
Fly ash (PF)	20	20	20

**Table 4:** Bulk density of the obtained aggregates after the sintering process.

Granulate/Aggregate	Bulk Density, kg/m^3^
Raw granulate G1	979
Aggregate G1 (sintered)	814
Raw granulate G2	983
Aggregate G2 (sintered)	846
Raw granulate G3	999
Aggregate G3 (sintered)	868

## Data Availability

The data presented in this study are available on request from the corresponding author.
